# Red blood cell distribution width to albumin ratio associates with prevalence and long-term diabetes mellitus prognosis: an overview of NHANES 1999–2020 data

**DOI:** 10.3389/fendo.2024.1362077

**Published:** 2024-07-24

**Authors:** Jie Liu, Xu Wang, Tian ye Gao, Qing Zhang, Sheng nan Zhang, Yuan yuan Xu, Wen qiang Yao, Zhen hua Yang, Hao jie Yan

**Affiliations:** ^1^ Nanjing University of Chinese Medicine, Nanjing, Jiangsu, China; ^2^ The Third Hospital of Guangxi University of Chinese Medicine, Liuzhou, Guangxi, China; ^3^ People’s Hospital of Chongqing Banan District, Chongqing, China; ^4^ Cangzhou Hospital of Integrated Traditional Chinese and Western Medicine, Cangzhou, Hebei, China; ^5^ Tianjin Nankai Hospital, Tianjin, China

**Keywords:** red blood cell distribution width to albumin ratio, NHANES, diabetes mellitus, prognosis, prevalence

## Abstract

**Background:**

Erythrocyte dysfunction is a characteristic of diabetes mellitus (DM). However, erythrocyte-associated biomarkers do not adequately explain the high prevalence of DM. Here, we describe red blood cell distribution width to albumin ratio (RAR) as a novel inflammatory biomarker for evaluating an association with DM prevalence and prognosis of all-cause mortality.

**Methods:**

Data analyzed in this study were extracted from the National Health and Nutrition Examination Survey (NHANES) 1999−2020. A total of 40,558 participants (non-DM and DM) were enrolled in the study; RAR quartiles were calibrated at Q1 [2.02,2.82] mL/g, Q2 (2.82,3.05] mL/g, Q3 (3.05,3.38] mL/g, and Q4 (3.38,12.08] mL/g. A total of 8,482 DM patients were followed (for a median of 84 months), of whom 2,411 died and 6,071 survived. The prevalence and prognosis associated with RAR and DM were analyzed; age and sex were stratified to analyze the prevalence of RAR in DM and the sensitivity of long-term prognosis.

**Results:**

Among non-DM (*n*=30,404) and DM (*n*=10,154) volunteers, DM prevalence in RAR quartiles was 8.23%, 15.20%, 23.92%, and 36.39%. The multivariable odds ratio (OR) was significant for RAR regarding DM, at 1.68 (95% CI 1.42, 1.98). Considering Q1 as a foundation, the Q4 OR was 2.57 (95% CI 2.11, 3.13). The percentages of DM morbidity varied across RAR quartiles for dead (*n*=2,411) and surviving (*n*=6,071) DM patients. Specifically, RAR quartile mortality ratios were 20.31%, 24.24%, 22.65%, and 29.99% (P<0.0001). The multivariable hazard ratio (HR) for RAR was 1.80 (95% CI 1.57, 2.05). Considering Q1 as a foundation, the Q4 HR was 2.59 (95% CI 2.18, 3.09) after adjusting for confounding factors. Sensitivity analysis revealed the HR of male DM patients to be 2.27 (95% CI 1.95, 2.64), higher than females 1.56 (95% CI 1.31, 1.85). DM patients who were 60 years of age or younger had a higher HR of 2.08 (95% CI1.61, 2.70) as compared to those older than 60 years, who had an HR of 1.69 (95% CI 1.47, 1.94). The HR of RAR in DM patients was optimized by a restricted cubic spline (RCS) model; 3.22 was determined to be the inflection point of an inverse L-curve. DM patients with a RAR >3.22 mL/g suffered shorter survival and higher mortality as compared to those with RAR ≤3.22 mL/g. OR and HR RAR values were much higher than those of regular red blood cell distribution width.

**Conclusions:**

The predictive value of RAR is more accurate than that of RDW for projecting DM prevalence, while RAR, a DM risk factor, has long-term prognostic power for the condition. Survival time was found to be reduced as RAR increased for those aged ≤60 years among female DM patients.

## 1 Introduction

Diabetes mellitus (DM) is now considered to be a global epidemic (1). Importantly, DM-related erythrocyte dysfunction is frequently seen in these patients and is pathologically characterized by three stages of severity; initially cholesterol clusters within the erythrocyte membrane (2), erythrocyte osmotic instability (3), and finally, decreased erythrocyte deformability (3). Erythrocyte dysfunction progresses along with the pathogenesis of DM. Increased erythrocyte nitric oxide and endothelial damage were reported in pre-diabetic subjects (4). In late DM, brittle erythrocytes become prone to rupture (5, 6). Although certain erythrocyte-related markers such as red blood cell distribution width (RDW) (5) and hemoglobin (6) are known to associate with DM, they fail to epidemiologically define the generally poor prognosis and highly prevalent nature of DM. For example, relevant odds (OR) and hazard (HR) ratios of RDW are only 1.16 (7) and 1.198 (8), respectively. Here, we evaluated for other indicators useful in detailing the epidemiology of DM.

Prior research has suggested other relevant indicators to be potentially capable of epidemiologically bridging erythrocyte-related markers and DM. RDW to albumin ratio (RAR), a novel inflammatory biomarker, is already widely applied in the setting of various illnesses. For example, RAR independently describes the all-cause mortality of heart failure (9), sepsis (10), and surgical burn wound management (11). Importantly, RAR is known to associate with various complications of DM such as diabetic retinopathy prevalence (12) and a poor prognosis of DM-related foot ulcers (13). However, the role of RAR in the epidemiology of DM itself remains unclear. Here, we integrate cross-sectional and prospective data obtained from over 40,000 subjects to explore the favorable predictive value of RAR for DM-related complications in the context of disease prevalence, prognosis, and all-cause mortality.

## 2 Methods

### 2.1 Participants and DM diagnosis

This study analyzed data originally compiled in the National Health and Nutrition Examination Survey (NHANES) public database between the years 1999 and 2020 (https://www.cdc.gov/nchs/nhanes/index.htm).

The diagnosis of DM was determined by the presence of five criteria (14), which included: i) physician confirmation of diabetes diagnosis, ii) glycohemoglobin levels equal to or greater than 6.5%, iii) fasting glucose ≥ 7.0 mmol/L, iv) random blood glucose≥ 11.1 mmol/L, and v) documented use of DM medication.

### 2.2 The mortality and follow-time definition

Data from the National Death Index supplemented NHANES data. As previously reported (15), DM mortality-related details (16) were matched to a unique NHANES identity number. Follow-up time was calculated from blood drawing to death or December 31, 2019.

### 2.3 Covariates

Other DM-related conditions were diagnosed using the medical conditions questionnaire (MCQ). Three criteria were considered for covariate filtering: i) demographics variables; ii) previously reported characteristics affecting DM; and iii) treatment-dependent clinical variables.

Medical history details including chronic kidney disease (CKD), chronic obstructive pulmonary disease (COPD), hypertension, arteriosclerotic cardiovascular disease (ASCVD), anemia and congestive heart failure (CHF) were considered to be covariates. Medical diagnoses were established based on accepted guidelines, such as those for hypertension (17). Four criteria were considered for COPD diagnosis, including a post-bronchodilator FEV1/FVC<0.7, a patient self-reported COPD diagnosis (MCQ160g and MCQ160p), a history of smoking and chronic respiratory disease in patients over 40 years of age, or a history of COPD medication use. Patients with a prior history of CHF, angina or stroke were diagnosed with atherosclerotic cardiovascular disease (ASCVD).

Clinical tests were classified as covariates. Biochemical indices considered in this study included blood levels of albumin (ALB), alanine transaminase (ALT), aspartate transaminase (AST), total calcium (Ca), bicarbonate (HCO3), gamma-glutamyl transferase (GGT), glucose (Glu), total protein (TP), triglycerides (TG), uric acid (UA), sodium (Na) and chloride (Cl).

Routine blood parameters considered in this study included percentages of basophils, lymphocytes, monocytes, segmented neutrophils and eosinophils (BaP, LymP, MonP, SegneP, and EoP, respectively), as well as counts of lymphocytes, monocytes, eosinophils, basophils and red blood cells (Lym, Mon, RBC, Eo, Ba, and RBC, respectively). Other hematologic parameters considered for analyses included hemoglobin (Hg), hematocrit (Hem), mean cell hemoglobin (MCH), mean cell hemoglobin concentration (MCHC), RDW, platelet count (Plt), mean platelet volume (MPV) and mean cell volume (MCV).

### 2.4 Statistical analyses

Statistical analyses were performed using R software (version 4.3.0). The nhanesR package (version 0.9.4.8) was used to glean clinical data from the NHANES database.

Student t-tests were adopted when continuous variables followed a Gaussian distribution on analysis of variance. Otherwise, the Mann-Whitney U test was applied. The chi-squared test was used to evaluate variable factors.

To better explore the relationship between DM prevalence and RAR stratification, we divided them into four groups (Q1, Q2, Q3, and Q4) according to the quartile data of RAR. The ranges of RAR (mL/g) in Q1-Q4 are [2.02,2.82], (2.82,3.05], (3.05,3.38], and (3.38,12.08], respectively.

Logistic regression was used to determine DM and healthy group OR, while Cox regression was used to determine HR. Both OR and HR were calculated with corresponding 95% confidence intervals (CIs). To better explore the relationship between RAR and DM, four models were employed in the adjustment. Model 1: adjusted with none; Model 2: adjusted with Sex and Age; Model 3: adjusted with Sex, Age, CKD, COPD, Hypertension, ASCVD, Anemia, CHF, Ethnicity; Model 4: adjusted with Age, Sex, CKD, COPD, Hypertension, ASCVD, Anemia, CHF, Ethnicity, LymP, SegneP, EoP, BaP, Lym, Mon, Eo, Ba, MCV, MCH, MCHC, Plt, MPV, ALT, AST, HCO3, GGT, Glu, TP, TG, UA, Na, and Cl.

Kaplan–Meier curves were utilized for survival analysis. Restricted cubic spline (RCS) analysis (18) was employed to filter for an optimal RAR threshold in predicting DM patient all-cause mortality.

## 3 Result

### 3.1 Basic information

**Figure 1:**
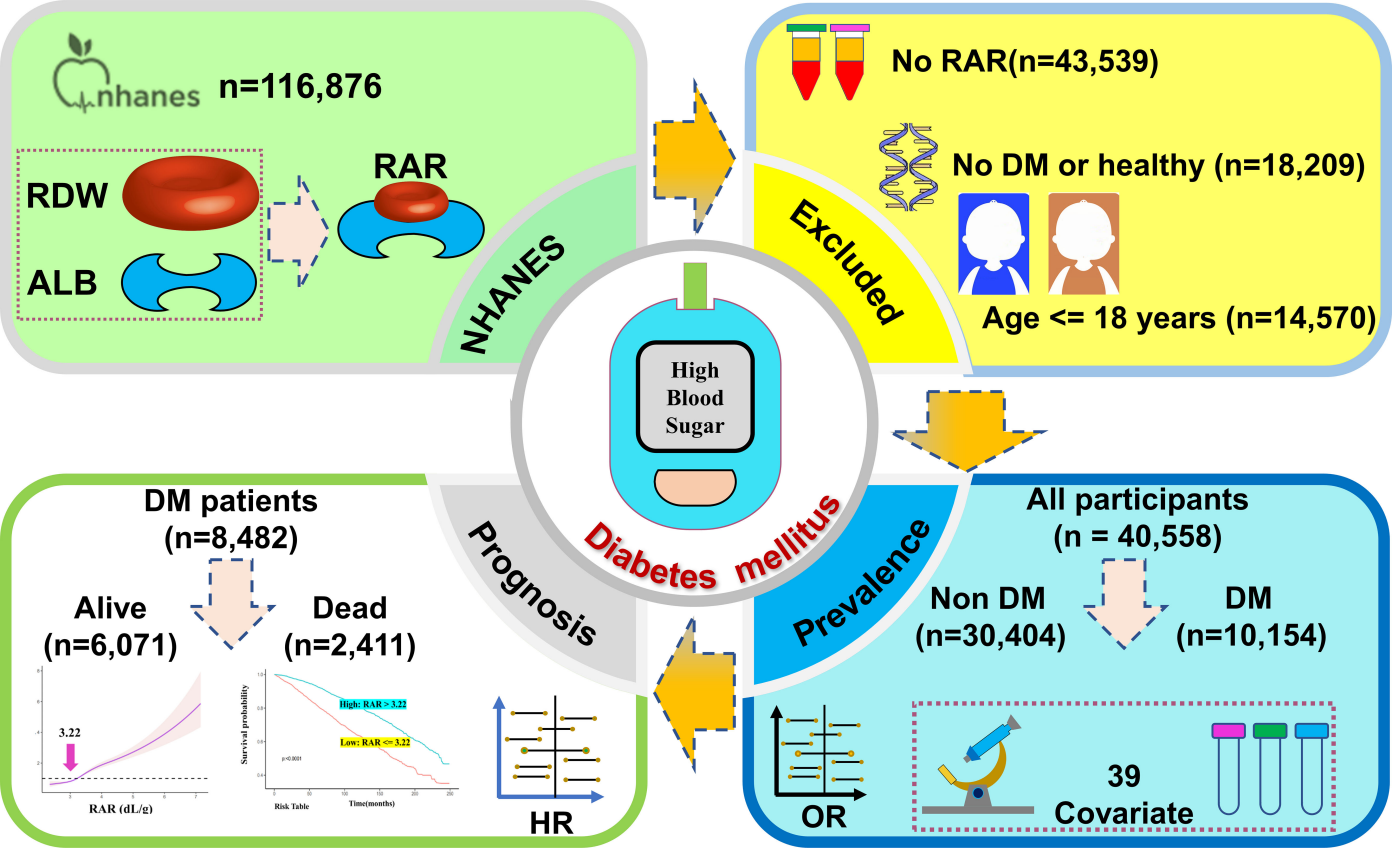
Study flow chart.

Subjects who were over 18 years of age and had complete RAR, demographic, clinical, and laboratory data available were included in analyses. Exclusion criteria were as follows: i) incomplete or unavailable RAR (*n*=43,539) or DM questionnaire (*n*=4) data; ii) individuals less than 18 years of age (*n*=14,570); or iii) diagnoses were unclear (*n*=18,205). A total of 40,558 individuals were ultimately included in this study. Of the 10,154 DM patients, 1,672 did not attend follow-up. As such, a total of 40,558 patients were included in DM prevalence analysis of DM, while 8,482 were included in all-cause mortality analysis (**Figure 1**).

As shown in **Table 1**, a total of 40,558 subjects were analyzed in this study as representative of 148,827,373 Americans, of whom 21,630 (53.33%) were females and 18,928 (46.67%) were males. The average age of subjects was 46.57 years, and 11,253 (27.75%) were older than 60 years of age. The 40,558 subjects were divided into non-DM (*n*=30,404) or DM (*n*=10,154) groups. The DM group had a higher RAR (3.31 ± 0.01) as compared to the non-DM group (3.02 ± 0.00). Only three of the 40 individuals had insignificant results, specifically concerning MonP, RBC, and Ca data. Among DM patients, there was a higher percentage of individuals with conditions such as CKD, COPD, hypertension, ASCVD, and CHF as compared to non-DM patients. Greater percentages of mild and moderate anemia were noted among DM patients.

**Table 1:** Basic demographic data of DM and non-DM subjects.

Variable	non-DM (*n*=30,404)	DM (*n*=10,154)	P_value
RAR, mL/g	3.02±0.00	3.31±0.01	< 0.0001
CKD, %	3239( 8.85)	4131(37.29)	< 0.0001
COPD, %	645(2.49)	590(7.24)	< 0.0001
Hypertension, %	7928(24.43)	7169(68.87)	< 0.0001
ASCVD, %	1570( 4.23)	2355(22.24)	< 0.0001
Anemia, %			< 0.0001
Mild	1614(3.65)	1232(9.16)	
Moderate	589(1.34)	401(3.00)	
Non-Anaemia	28152(94.91)	8510(87.80)	
Severe	49(0.10)	11(0.04)	
CHF, %	414(1.00)	910(8.14)	< 0.0001
Ethnicity, %			< 0.0001
Mexican American	5266(8.02)	1964(9.31)	
Non-Hispanic Black	5708( 9.48)	2519(13.87)	
Non-Hispanic White	14171(70.32)	3620(61.93)	
Other Hispanic	2406(5.83)	977(6.15)	
Other Race	2853(6.35)	1074(8.74)	
Sex, (Male), %	13656(45.69)	5272(51.59)	< 0.0001
Age, year	41.32±0.19	59.24±0.22	< 0.0001
LymP, %	30.23±0.07	28.64±0.15	< 0.0001
MonP, %	7.94±0.02	7.89±0.03	0.12
SegneP, %	58.47±0.08	59.88±0.15	< 0.0001
EoP, %	2.70±0.01	2.93±0.03	< 0.0001
BaP, %	0.71±0.00	0.73±0.01	< 0.001
Lym, 1000 cells/μL	2.12±0.01	2.19±0.02	< 0.0001
Mon, 1000 cells/μL	0.56±0.00	0.60±0.00	< 0.0001
Eo, 1000 cells/μL	0.19±0.00	0.22±0.00	< 0.0001
Ba, 1000 cells/μL	0.04±0.00	0.05±0.00	< 0.0001
RBC, million cells/μL	4.68±0.01	4.69±0.01	0.21
Hg, g/dl	14.29±0.02	14.09±0.03	< 0.0001
Hem, %	42.01±0.06	41.69±0.07	< 0.0001
MCV, fL	89.90±0.06	89.11±0.09	< 0.0001
MCH, pg	30.59±0.03	30.12±0.04	< 0.0001
MCHC, g/cL	34.01±0.02	33.78±0.03	< 0.0001
RDW, %	12.89±0.01	13.52±0.02	< 0.0001
Plt, 1000 cells/μL	254.67±0.70	246.70±1.21	< 0.0001
MPV, fL	8.17±0.01	8.30±0.02	< 0.0001
ALB, g/dL	4.31±0.00	4.13±0.01	< 0.0001
ALT, U/L	23.92±0.13	27.38±0.34	< 0.0001
AST, U/L	24.28±0.11	25.99±0.26	< 0.0001
Ca, mg/dL	9.42±0.01	9.41±0.01	0.21
HCO3, mmol/L	24.74±0.05	24.97±0.05	< 0.0001
GGT, U/L	25.56±0.26	37.79±0.80	< 0.0001
Glu, mg/dL	87.76±0.10	146.42±0.89	< 0.0001
TP, g/dL	7.17±0.01	7.13±0.01	< 0.0001
TG, mg/dL	134.02±1.02	198.56±2.91	< 0.0001
UA, mg/dL	5.18±0.01	5.72±0.02	< 0.0001
Na, mmol/L	139.33±0.06	139.05±0.09	< 0.0001
Cl, mmol/L	103.27±0.06	102.03±0.07	< 0.0001

The RAR quartiles (RARQ) were described. General RAR quartile data are summarized in **Table 2**. The incidence of DM among RARQ was 8.23%, 15.20%, 23.92%, and 36.39%, respectively; DM prevalence increased as RAR increased. Elevated RAR was associated with a significantly greater proportion of male and older patients. Furthermore, males had a higher incidence of DM and generally lower RAR as compared to females (**Supplementary Table S1**). A greater percentage of older individuals had DM and a higher RAR (**Supplementary Table S2**). As shown in **Table 1**, the incidence of CKD, COPD, hypertension, ASCVD, and CHF increased with rising RARQ. The percentage of patients who were Mexican Americans, non-Hispanic blacks, and other Hispanics increased as RAR increased, as the percentage of non-Hispanic whites decreased.

**Table 2:** RAR quartile data.

Variables	Q1 (*n*=11,489)	Q2 (*n*=10,105)	Q3 (*n*=9,484)	Q4 (*n*=9,480)	P_value
**RAR rang, mL/g**	**[2.02,2.82]**	**(2.82,3.05]**	**(3.05,3.38]**	**(3.38,12.08]**	
DM, %	1320( 8.23)	2084(15.20)	2813(23.92)	3937(36.39)	< 0.0001
RAR, dL/g	2.67±0.00	2.97±0.00	3.23±0.00	3.86±0.01	< 0.0001
CKD, %	1063( 7.42)	1449(11.49)	1959(17.53)	2899(27.16)	< 0.0001
COPD, %	194(1.82)	278(3.37)	328(4.05)	435(5.97)	< 0.0001
Hypertension, %	2846(23.35)	3545(31.84)	4026(37.47)	4680(46.12)	< 0.0001
ASCVD, %	498( 3.38)	752( 6.18)	1084( 9.55)	1591(15.62)	< 0.0001
Anemia, %					< 0.0001
Mild	151( 0.83)	325( 2.30)	653( 4.90)	1717(15.70)	
Moderate	10(0.06)	37(0.22)	72(0.58)	871(8.40)	
Non-Anaemia	11328(99.10)	9743(97.48)	8759(94.53)	6832(75.36)	
Severe	0(0.00)	0(0.00)	0(0.00)	60(0.54)	
CHF, %	103(0.62)	193(1.55)	318(2.66)	710(6.48)	< 0.0001
Ethnicity, %					< 0.0001
Mexican American	2409(8.13)	1864(8.27)	1564(8.28)	1393(8.48)	
Non-Hispanic Black	1259( 4.92)	1682( 8.62)	2130(12.26)	3156(21.19)	
Non-Hispanic White	5896(74.90)	4637(70.36)	3969(66.82)	3289(56.48)	
Other Hispanic	849(5.53)	850(5.74)	899(6.11)	785(6.54)	
Other Race	1076(6.52)	1072(7.01)	922(6.53)	857(7.31)	
Sex, (Male), %	7128(60.69)	4943(47.34)	3868(38.34)	2989(28.36)	< 0.0001
Age, year	38.94±0.26	44.93±0.26	48.37±0.33	50.79±0.29	< 0.0001
LymP, %	30.51±0.10	30.44±0.11	29.81±0.14	28.20±0.15	< 0.0001
MonP, %	7.93±0.03	7.96±0.03	7.89±0.03	7.96±0.03	0.23
SegneP, %	58.24±0.10	58.18±0.12	58.87±0.16	60.40±0.17	< 0.0001
EoP, %	2.71±0.02	2.77±0.02	2.77±0.03	2.76±0.02	0.18
BaP, %	0.67±0.01	0.71±0.01	0.74±0.01	0.76±0.01	< 0.0001
Lym, 1000 cells/μL	2.12±0.01	2.14±0.01	2.18±0.02	2.11±0.01	0.002
Mon, 1000 cells/μL	0.55±0.00	0.56±0.00	0.57±0.00	0.60±0.00	< 0.0001
Eo, 1000 cells/μL	0.19±0.00	0.20±0.00	0.20±0.00	0.21±0.00	< 0.0001
Ba, 1000 cells/μL	0.04±0.00	0.04±0.00	0.05±0.00	0.05±0.00	< 0.0001
RBC, million cells/μL	4.80±0.01	4.69±0.01	4.63±0.01	4.51±0.01	< 0.0001
Hg, g/dl	14.88±0.03	14.41±0.02	14.05±0.03	13.01±0.03	< 0.0001
Hem, %	43.49±0.07	42.27±0.07	41.44±0.08	38.98±0.09	< 0.0001
MCV, fL	90.85±0.07	90.31±0.07	89.65±0.09	86.84±0.11	< 0.0001
MCH, pg	31.10±0.03	30.78±0.03	30.40±0.04	29.00±0.05	< 0.0001
MCHC, g/cL	34.23±0.03	34.08±0.03	33.90±0.03	33.35±0.03	< 0.0001
RDW, %	12.22±0.01	12.76±0.01	13.25±0.01	14.66±0.03	< 0.0001
Plt, 1000 cells/μL	251.69±0.87	251.26±1.03	252.22±1.04	260.54±1.49	< 0.0001
MPV, fL	8.13±0.01	8.18±0.02	8.24±0.02	8.28±0.02	< 0.0001
ALB, g/dL	4.59±0.00	4.30±0.00	4.11±0.00	3.82±0.00	< 0.0001
ALT, U/L	26.40±0.21	25.05±0.25	23.38±0.23	21.51±0.24	< 0.0001
AST, U/L	25.44±0.17	24.73±0.19	23.91±0.20	23.52±0.20	< 0.0001
Ca, mg/dL	9.58±0.01	9.43±0.01	9.33±0.01	9.20±0.01	< 0.0001
HCO3, mmol/L	24.87±0.06	24.77±0.05	24.78±0.05	24.61±0.07	0.01
GGT, U/L	26.51±0.35	27.36±0.56	27.51±0.57	31.45±0.76	< 0.0001
Glu, mg/dL	92.02±0.29	96.60±0.43	102.79±0.51	109.08±0.63	< 0.0001
TP, g/dL	7.37±0.01	7.15±0.01	7.03±0.01	6.92±0.01	< 0.0001
TG, mg/dL	142.68±1.74	146.57±1.72	149.08±2.19	146.99±1.84	0.07
UA, mg/dL	5.39±0.01	5.24±0.02	5.18±0.02	5.22±0.02	< 0.0001
Na, mmol/L	139.30±0.06	139.25±0.06	139.33±0.08	139.23±0.12	0.2
Cl, mmol/L	102.87±0.07	103.22±0.07	103.15±0.06	103.00±0.07	< 0.0001

### 3.2 DM prevalence in RAR

Logistic regression data are presented in **Supplementary Table S3** detailing RAR, RARQ, RDW, and ALB. Three different combinations of variables were used to adjust the model. Model 1 was an unadjusted univariate logistic regression model. Model 2 was just adjusted for age and sex. Model 3 encompassed Model 2, ethnicity, and medical history data (e.g. CKD, COPD). Model 4 corrected for more variables than other models, encompassing Model 3 and laboratory data (e.g. UA, Cl). Interestingly, OR values of RAR remained significant on univariate analysis and the three adjusted models. The ORs for the three adjusted models were 2.61 (95% CI 2.42, 2.82), 2.20 (95% CI 1.98, 2.45) and 1.68 (95% CI 1.42, 1.98). To determine the prevalence of DM in different RAR stratifications, Q1 served as a reference for the aforementioned multivariate analyses (**Table 3**). In Model 4, the RAR quartile ORs were 1.32 for Q2, 1.74 for Q3, and 2.57 for Q4. In conclusion, as RAR quartile progressed, relevant OR increased, indicating that RAR is a risk for DM prevalence.

**Table 3:** Logistic regression for RAR, RARQ, RDW, and ALB.

Variables	DM							
	OR (95% CI)^a^	P_value	OR (95% CI)^b^	P_value	OR (95% CI)^c^	P_value	OR (95% CI)^d^	P_value
RAR	3.05(2.85,3.27)	<0.0001	2.61(2.42,2.82)	<0.0001	2.20(1.98,2.45)	<0.0001	1.68(1.42, 1.98)	<0.0001
RARQ								
Q1	ref	ref	ref	ref	ref	ref	ref	ref
Q2	2.00(1.80,2.22)	<0.0001	1.50(1.36,1.67)	<0.0001	1.41(1.26,1.58)	<0.0001	1.32(1.13, 1.53)	<0.001
Q3	3.51(3.18,3.87)	<0.0001	2.38(2.14,2.66)	<0.0001	2.08(1.85,2.33)	<0.0001	1.74(1.48, 2.06)	<0.0001
Q4	6.38(5.76,7.07)	<0.0001	4.47(4.01,4.98)	<0.0001	3.30(2.90,3.74)	<0.0001	2.57(2.11, 3.13)	<0.0001
RDW	1.42(1.38,1.46)	<0.0001	1.31(1.27,1.35)	<0.0001	1.22(1.17,1.26)	<0.0001	1.14(1.09, 1.20)	<0.0001
ALB	0.25(0.23,0.28)	<0.0001	0.28(0.25,0.31)	<0.0001	0.37(0.33,0.42)	<0.0001	0.50(0.41, 0.61)	<0.0001

a Model 1: unadjusted; b Model 2: adjusted with Sex and Age; c Model 3: adjusted with Sex, Age, CKD, COPD, Hypertension, ASCVD, Anemia, CHF, Ethnicity; d Model 4: adjusted with Age, Sex, CKD, COPD, Hypertension, ASCVD, Anemia, CHF, Ethnicity, LymP, SegneP, EoP, BaP, Lym, Mon, Eo, Ba, MCV, MCH, MCHC, Plt, MPV, ALT, AST, HCO3, GGT, Glu, TP, TG, UA, Na, and Cl.

Importantly, RAR OR was consistently greater than RDW OR. For instance, in the adjusted Model 4, RAR OR, and RDW OR were 1.68 and 1.14, respectively. Meanwhile, ALB was found to be a protective factor for DM with an OR of 0.50 (95% CI 0.41, 0.61). Although RAR and RDW are independent risk factors for DM, the greater RAR OR highlights its better predictive value for DM. As such, the clinical value of RAR data is superior to that of RDW for determining DM prevalence.

### 3.3 Sensitivity analysis for age and sex

The clinical value of RAR for DM in various sex and age stratifications was assessed; details are summarized in **Supplementary Tables S4**-**5**. The incidence of DM was found to have been significantly associated with age and gender stratifications in Models 1–3, although in Model 4 the association was not significant. Adjusted Models 2 and 3 revealed that RAR was significantly associated with DM in females (OR: 2.23; 95% CI 1.85, 2.68) and males (OR: 2.13; (95% CI 1.85, 2.46). The OR values of female patients were greater than those of male patients, suggesting a higher sensitivity for females. The RAR OR of patients aged ≤60 years (2.65; 95% CI 2.28, 3.07) was significantly higher than among those older than 60 years (1.76; 95% CI 1.51, 2.05), which indicated a higher sensitivity for participants aged ≤60 years. However, the OR values for sex and age levels were insignificant on adjustment of Model 4 (**Supplementary Tables S4**-**5**).

### 3.4 Survival analysis

Due to the absence of follow-up data, 1,672 DM patients were excluded from survival analysis while a total of 8,482 DM patients were finally included. Throughout the follow-up period, 2,411 DM patients died; 6,071 survived (**Table 4**). The duration of follow-up ranged from one to 249 months. The RAR found in deceased DM patients (3.37 ± 0.02) was much greater than that found in surviving DM patients (3.25 ± 0.01). The deceased DM patients were of significantly greater age, although patient sex was not found to be significant. Furthermore, a greater percentage of deceased DM patients also suffered CKD, COPD, hypertension, ASCVD, CHF, and/or anemia, according to medical records.

**Table 4:** Primary characteristics of deceased and surviving DM patients.

Variable	DM patients alive (*n*=6,071)	DM patients alive (*n*=2,411)	P_value
RAR, mL/g	3.25±0.01	3.37±0.02	< 0.0001
CKD, %	1969(29.53)	1489(60.55)	< 0.0001
COPD, %	333( 5.86)	257(11.40)	< 0.0001
Hypertension, %	4129(65.59)	1934(80.32)	< 0.0001
ASCVD, %	1050(16.68)	918(37.85)	< 0.0001
Anemia, %			< 0.0001
Mild	597( 7.15)	421(15.16)	
Moderate	192(2.51)	133(4.42)	
Non-Anaemia	5277(90.30)	1854(80.38)	
Severe	6(0.04)	3(0.04)	
CHF, %	334( 5.03)	422(17.55)	< 0.0001
Ethnicity, %			< 0.0001
Mexican American	1333(10.63)	403( 5.28)	
Non-Hispanic Black	1483(14.24)	573(13.45)	
Non-Hispanic White	1892(59.11)	1216(72.38)	
Other Hispanic	667(6.68)	123(3.77)	
Other Race	697(9.33)	96(5.12)	
Sex, (Male), %	3023(50.92)	1370(52.85)	0.26
Age, year	56.16±0.26	68.18±0.34	< 0.0001
LymP, %	29.30±0.18	26.59±0.26	< 0.0001
MonP, %	7.73±0.04	8.22±0.07	< 0.0001
SegneP, %	59.39±0.19	61.53±0.28	< 0.0001
EoP, %	2.91±0.04	3.02±0.05	0.15
BaP, %	0.73±0.01	0.69±0.01	< 0.001
Lym, 1000 cells/μL	2.22±0.02	2.06±0.04	< 0.001
Mon, 1000 cells/μL	0.58±0.00	0.62±0.01	< 0.0001
Eo, 1000 cells/μL	0.22±0.00	0.23±0.00	0.23
Ba, 1000 cells/μL	0.05±0.00	0.05±0.00	0.02
RBC, million cells/μL	4.74±0.01	4.53±0.01	< 0.0001
Hg, g/dl	14.18±0.03	13.85±0.05	< 0.0001
Hem, %	41.90±0.08	40.99±0.14	< 0.0001
MCV, fL	88.63±0.10	90.79±0.15	< 0.0001
MCH, pg	29.99±0.05	30.69±0.06	< 0.0001
MCHC, g/cL	33.81±0.03	33.79±0.04	0.56
RDW, %	13.42±0.03	13.58±0.04	< 0.001
Plt, 1000 cells/μL	249.00±1.62	241.53±1.98	0.004
MPV, fL	8.31±0.02	8.22±0.03	0.01
ALB, g/dL	4.17±0.01	4.08±0.01	< 0.0001
ALT, U/L	28.42±0.38	25.68±0.83	0.002
AST, U/L	26.36±0.32	26.85±0.55	0.42
Ca, mg/dL	9.42±0.01	9.46±0.01	0.004
HCO3, mmol/L	24.89±0.07	24.88±0.09	0.93
GGT, U/L	35.37±0.74	44.71±2.66	< 0.001
Glu, mg/dL	146.09±1.10	150.56±1.95	0.05
TP, g/dL	7.13±0.01	7.17±0.02	0.06
TG, mg/dL	201.33±3.99	198.67±4.89	0.68
UA, mg/dL	5.62±0.03	6.11±0.05	< 0.0001
Na, mmol/L	138.92±0.11	138.81±0.09	0.39
Cl, mmol/L	102.32±0.08	102.00±0.11	0.01

Relevant details of RARQ are summarized in **Table 5**. As RAR values increased, so did the prevalence of CKD, COPD, hypertension, ASCVD, and CHF. The fatality rates of DM patients among RARQ were 20.31%, 24.24%, 22.65% and 29.99%, respectively (*P*<0.001). Among the RARQ, Q4 had the highest HR value.

**Table 5:** Basic RAR quartile data of DM patients who were followed-up.

Variable	Q1 (*n*=1,275)	Q2 (*n*=1,898)	Q3 (*n*=2,359)	Q4 (*n*=2,951)	P_value
**RAR rang, mL/g**	**[2.02,2.82]**	**(2.82,3.05]**	**(3.05,3.38]**	**(3.38,12.08]**	
Mortality, %	307(20.31)	510(24.24)	627(22.65)	967(29.99)	< 0.0001
RAR, mL/g	2.71±0.00	2.97±0.00	3.23±0.00	3.85±0.01	< 0.0001
CKD, %	399(27.71)	625(29.64)	937(36.68)	1497(48.62)	< 0.0001
COPD, %	47( 4.03)	96( 6.06)	160( 6.93)	287(10.15)	< 0.0001
Hypertension, %	787(57.61)	1320(69.05)	1681(69.10)	2275(75.92)	< 0.0001
ASCVD, %	186(12.08)	366(19.37)	556(22.99)	860(28.35)	< 0.0001
Anemia, %					< 0.0001
Mild	46( 2.17)	100( 4.05)	215( 6.60)	657(18.98)	
Moderate	3(0.11)	13(0.38)	29(0.75)	280(8.46)	
Non-Anaemia	1226(97.73)	1785(95.57)	2115(92.65)	2005(72.43)	
Severe	0(0.00)	0(0.00)	0(0.00)	9(0.13)	
CHF, %	39( 2.62)	105( 5.37)	182( 7.13)	430(14.11)	< 0.0001
Ethnicity, %					< 0.0001
Mexican American	367(10.89)	449( 9.77)	473( 9.29)	447( 8.08)	
Non-Hispanic Black	129( 4.87)	320( 9.55)	562(13.58)	1045(22.78)	
Non-Hispanic White	522(66.67)	741(65.96)	881(64.11)	964(55.99)	
Other Hispanic	111(6.44)	180(5.98)	250(6.15)	249(5.50)	
Other Race	146(11.13)	208( 8.74)	193( 6.86)	246( 7.66)	
Sex, (Male), %	848(68.09)	1104(59.50)	1177(48.53)	1264(38.89)	< 0.0001
Age, year	55.31±0.52	58.76±0.39	59.88±0.42	60.91±0.34	< 0.0001
LymP, %	30.38±0.36	29.41±0.23	28.68±0.33	27.05±0.25	< 0.0001
MonP, %	7.91±0.08	7.85±0.08	7.77±0.06	7.90±0.07	0.41
SegneP, %	58.26±0.38	59.15±0.26	59.90±0.33	61.42±0.27	< 0.0001
EoP, %	2.83±0.06	2.93±0.06	2.99±0.07	2.95±0.05	0.28
BaP, %	0.67±0.01	0.72±0.02	0.73±0.01	0.75±0.01	< 0.0001
Lym, 1000 cells/μL	2.22±0.05	2.17±0.02	2.21±0.04	2.14±0.02	0.22
Mon, 1000 cells/μL	0.57±0.01	0.58±0.01	0.59±0.01	0.62±0.01	< 0.0001
Eo, 1000 cells/μL	0.21±0.01	0.22±0.01	0.23±0.01	0.23±0.00	< 0.001
Ba, 1000 cells/μL	0.04±0.00	0.05±0.00	0.05±0.00	0.06±0.00	< 0.0001
RBC, million cells/μL	4.82±0.02	4.74±0.02	4.68±0.02	4.57±0.01	< 0.0001
Hg, g/dl	14.98±0.06	14.55±0.04	14.18±0.04	13.21±0.05	< 0.0001
Hem, %	43.66±0.17	42.69±0.12	41.86±0.13	39.67±0.14	< 0.0001
MCV, fL	90.80±0.20	90.26±0.13	89.58±0.14	87.10±0.17	< 0.0001
MCH, pg	31.16±0.08	30.75±0.05	30.34±0.06	29.02±0.07	< 0.0001
MCHC, g/cL	34.31±0.06	34.07±0.04	33.86±0.03	33.29±0.04	< 0.0001
RDW, %	12.30±0.02	12.79±0.02	13.32±0.02	14.70±0.04	< 0.0001
Plt, 1000 cells/μL	243.68±2.66	243.18±2.17	245.00±2.17	253.83±2.38	0.002
MPV, fL	8.18±0.04	8.24±0.03	8.34±0.03	8.33±0.03	0.002
ALB, g/dL	4.54±0.01	4.30±0.01	4.12±0.01	3.84±0.01	< 0.0001
ALT, U/L	31.42±0.75	29.73±0.61	28.08±0.76	23.96±0.50	< 0.0001
AST, U/L	28.02±0.59	27.23±0.42	26.42±0.56	25.16±0.44	< 0.001
Ca, mg/dL	9.61±0.02	9.50±0.01	9.42±0.01	9.28±0.01	< 0.0001
HCO3, mmol/L	24.84±0.09	24.79±0.08	24.79±0.08	25.08±0.08	0.01
GGT, U/L	38.42±1.50	37.38±1.49	35.86±1.98	39.16±1.33	0.54
Glu, mg/dL	145.66±2.72	145.71±1.88	148.47±1.68	148.02±1.51	0.71
TP, g/dL	7.39±0.02	7.22±0.02	7.09±0.02	7.00±0.01	< 0.0001
TG, mg/dL	230.28±11.14	206.45± 5.44	199.54± 5.88	181.25± 3.92	< 0.0001
UA, mg/dL	5.63±0.05	5.69±0.05	5.66±0.05	5.90±0.04	< 0.001
Na, mmol/L	138.72±0.12	138.72±0.10	138.93±0.11	139.09±0.15	0.09
Cl, mmol/L	101.95±0.14	102.22±0.13	102.47±0.10	102.22±0.11	0.02

Cox regression analysis data relevant to RAR, RARQ, RDW, and ALB are shown in **Table 6**. As with logistic regression models, three models with multiple covariates were utilized to adjust for RAR HR, which remained significant in Model 4, at 1.80 (95% CI 1.57, 2.05). The Q2, Q3 and Q4 HR values remained significant even on an adjusted Model 4 (**Table 6**). The optimal HR was found in Q4, at 2.59 (95% CI 2.18, 3.09). As the RARQ advanced, RAR OR values increased, and DM prognosis became poorer. As such, RAR was found to be an independent risk factor for DM prognosis.

**Table 6:** Single and multiple variable regulation of the Cox regression model for RAR, RARQ, RDW, and ALB.

Variables	DM mortality							
	HR (95% CI)^a^	P_value	HR (95% CI)^b^	P_value	HR (95% CI)^c^	P_value	HR (95% CI)^d^	P_value
RAR	2.03(1.87,2.20)	<0.0001	2.05(1.88,2.23)	<0.0001	1.79(1.62,1.99)	<0.0001	1.80(1.57,2.05)	<0.0001
RARQ								
Q1	ref	ref	ref	ref	ref	ref	ref	ref
Q2	1.57(1.32,1.86)	<0.0001	1.36(1.16,1.60)	<0.001	1.32(1.12,1.56)	0.001	1.38(1.17,1.62)	<0.0001
Q3	1.81(1.52,2.17)	<0.0001	1.59(1.36,1.85)	<0.0001	1.43(1.23,1.67)	<0.0001	1.57(1.35,1.82)	<0.0001
Q4	3.37(2.86,3.99)	<0.0001	2.99(2.58,3.47)	<0.0001	2.29(1.93,2.71)	<0.0001	2.59(2.18,3.09)	<0.0001
RDW	1.23(1.19,1.27)	<0.0001	1.22(1.18,1.26)	<0.0001	1.16(1.11,1.21)	<0.0001	1.16(1.10,1.22)	<0.0001
ALB	0.40(0.35,0.46)	<0.0001	0.36(0.31,0.42)	<0.0001	0.49(0.41,0.57)	<0.0001	0.45(0.37,0.55)	<0.0001

a Model 1: unadjusted; b Model 2: adjusted with Sex and Age; c Model 3: adjusted with Sex, Age, CKD, COPD, Hypertension, ASCVD, Anemia, CHF, Ethnicity; d Model 4: adjusted with Age, Sex, CKD, COPD, Hypertension, ASCVD, Anemia, CHF, Ethnicity, LymP, SegneP, EoP, BaP, Lym, Mon, Eo, Ba, MCV, MCH, MCHC, Plt, MPV, ALT, AST, HCO3, GGT, Glu, TP, TG, UA, Na, and Cl.

Moreover, RAR HR was greater than RDW HR both in single or adjusted Cox regression. Although RAR HR values in the unadjusted and three adjusted models were 2.03, 2.05, 1.79 and 1.80, RDW HR values were 1.23, 1.22, 1.16 and 1.16, respectively. The RAR and RDW HR values in the adjusted Model 4 were 1.80 and 1.16, respectively. Thus, the RAR HR was found to possess superior predictive value for long-term DM prognosis as compared to RDW.

### 3.5 Sensitivity analysis for prognosis

To evaluate the HR sensitivity of sex and age in relation to RAR among DM patients, stratified Cox regression analysis was performed for DM patients of different ages and genders (**Table 7**). In adjusted Model 4, DM male patients had a higher HR (2.27; 95% CI 1.95, 2.64) as compared to females (1.56; 95% CI 1.31, 1.85). For RARQ and considering Q1 as a reference, adjusted Q4 for males (3.08; 95% CI 2.46, 3.85) was significantly higher as compared to females (2.19; 95% CI 1.64, 2.92). As such, male DM patients suffered a greater risk of poor prognosis when RAR values increased.

**Table 7:** Cox regression of DM patient data for RAR with stratification of sex.

Sex	Variables	DM							
		HR (95% CI)^a^	P-value	HR (95% CI)^b^	P-value	HR (95% CI)^c^	P-value	HR (95% CI)^d^	P-value
Male	RAR	2.48(2.17,2.84)	<0.0001	2.20(1.96,2.48)	<0.0001	1.98(1.73,2.27)	<0.0001	2.27(1.95, 2.64)	<0.0001
	Q1	ref	ref	ref	ref	ref	ref	ref	ref
	Q2	1.57(1.26,1.96)	<0.0001	1.37(1.11,1.68)	0.003	1.37(1.11,1.70)	0.003	1.46(1.18, 1.81)	<0.001
	Q3	2.16(1.68,2.77)	<0.0001	1.58(1.26,1.96)	<0.0001	1.47(1.17,1.85)	0.001	1.64(1.31, 2.06)	<0.0001
	Q4	4.88(3.93,6.06)	<0.0001	3.33(2.73,4.06)	<0.0001	2.49(2.01,3.09)	<0.0001	3.08(2.46, 3.85)	<0.0001
Female	RAR	1.79(1.61,1.98)	<0.0001	1.93(1.71,2.19)	<0.0001	1.64(1.43,1.89)	<0.0001	1.56(1.31,1.85)	<0.0001
	Q1	ref	ref	ref	ref	ref	ref	ref	ref
	Q2	1.59(1.18,2.15)	0.002	1.35(1.03,1.78)	0.03	1.22(0.92,1.61)	0.16	1.26(0.97,1.65)	0.09
	Q3	1.62(1.21,2.16)	0.001	1.63(1.24,2.14)	<0.001	1.38(1.06,1.80)	0.02	1.52(1.17,1.97)	0.001
	Q4	2.74(2.10,3.57)	<0.0001	2.77(2.17,3.55)	<0.0001	2.10(1.61,2.75)	<0.0001	2.19(1.64,2.92)	<0.0001

a Model 1: unadjusted; b Model 2: adjusted with Age; c Model 3: adjusted Age, CKD, COPD, Hypertension, ASCVD, Anemia, CHF, Ethnicity; d Model 4: adjusted with Age, CKD, COPD, Hypertension, ASCVD, Anemia, CHF, Ethnicity, LymP, SegneP, EoP, BaP, Lym, Mon, Eo, Ba, MCV, MCH, MCHC, Plt, MPV, ALT, AST, HCO3, GGT, Glu, TP, TG, UA, Na, and Cl.

As shown in **Table 8**, DM patients aged ≤60 years had a higher HR (2.08; 95% CI 1.61, 2.70) as compared to those older than 60 years (1.69; 95% CI 1.47, 1.94). The RAR HR for Q4 in DM patients aged ≤60 years (4.06; 95% CI 2.54, 6.49) was significantly greater than for those older than 60 years (2.06; 95% CI 1.70, 2.50). Poor prognosis was more prevalent among DM patients ≤60 years old as compared to >60 when RAR was increased.

**Table 8:** Cox regression of DM patient data for RAR with stratification of age.

Age	Variables	DM							
		HR (95% CI)^a^	P-value	HR (95% CI)^b^	P-value	HR (95% CI)^c^	P-value	HR (95% CI)^d^	P-value
>60	RAR	2.04(1.86,2.25)	<0.0001	2.06(1.86,2.27)	<0.0001	1.68(1.50,1.89)	<0.0001	1.69(1.47,1.94)	<0.0001
	Q1	ref	ref	ref	ref	ref	ref	ref	ref
	Q2	1.24(1.02,1.52)	0.03	1.26(1.04,1.54)	0.02	1.17(0.97,1.41)	0.11	1.12(0.92, 1.37)	0.26
	Q3	1.57(1.27,1.93)	<0.001	1.61(1.31,1.98)	<0.0001	1.36(1.13,1.63)	0.001	1.38(1.15, 1.66)	<0.001
	Q4	2.83(2.36,3.38)	<0.0001	2.94(2.46,3.52)	<0.0001	1.94(1.61,2.36)	<0.0001	2.06(1.70, 2.50)	<0.0001
<=60	RAR	2.02(1.73,2.36)	<0.0001	2.28(1.95,2.67)	<0.0001	2.07(1.73,2.48)	<0.0001	2.08(1.61, 2.70)	<0.0001
	Q1	ref	ref	ref	ref	ref	ref	ref	ref
	Q2	2.02(1.34,3.04)	<0.001	2.08(1.38,3.13)	<0.001	1.85(1.21,2.83)	0.005	2.04(1.33, 3.11)	0.001
	Q3	1.80(1.23,2.62)	0.002	2.20(1.49,3.23)	<0.0001	1.87(1.23,2.85)	0.004	2.01(1.29, 3.14)	0.002
	Q4	3.76(2.58,5.48)	<0.0001	5.00(3.45,7.23)	<0.0001	3.50(2.36,5.20)	<0.0001	4.06(2.54, 6.49)	<0.0001

a Model 1: unadjusted; b Model 2: adjusted with Sex; c Model 3: adjusted with Sex, CKD, COPD, Hypertension, ASCVD, Anemia, CHF, Ethnicity; d Model 4: adjusted with Sex, CKD, COPD, Hypertension, ASCVD, Anemia, CHF, Ethnicity, LymP, SegneP, EoP, BaP, Lym, Mon, Eo, Ba, MCV, MCH, MCHC, Plt, MPV, ALT, AST, HCO3, GGT, Glu, TP, TG, UA, Na, and Cl.

### 3.6 Survival analysis

**Figure 2:**
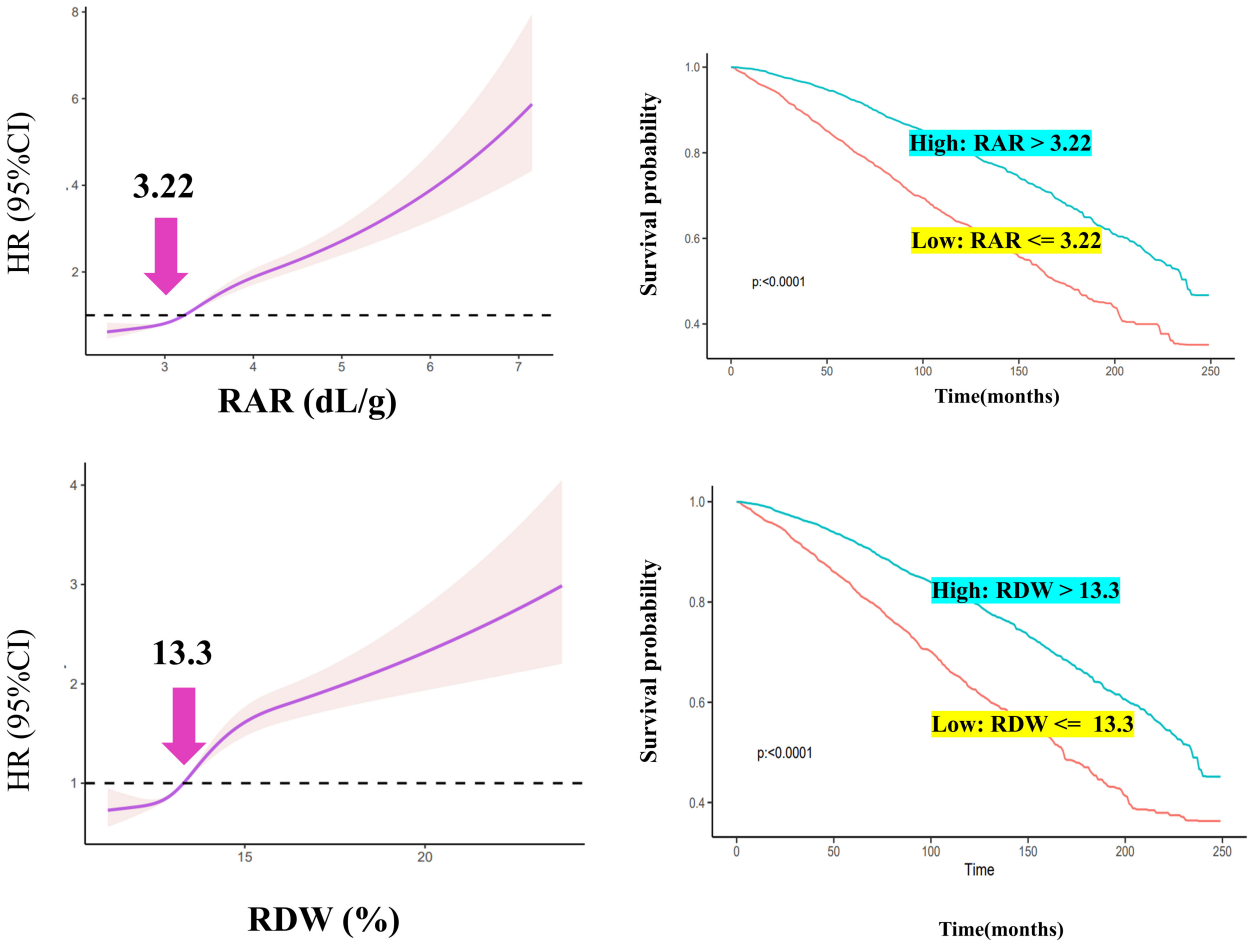
RCS analysis and Kaplan–Meier curve construction.

RCS was used to filter for optimal RAR and RDW values (**Figure 2A**). In DM patients, RDW and RAR were associated with a poor prognosis; HR values increased as RAR and RDW increased (**Figure 2A**). The inflection points of RAR and RDW were 3.22 and 13.3, respectively.

High- and low-expression groups were established based on the optimal inflection point. DM patients with RAR >3.22 were categorized into a high RAR group; those with RDW >13.3 were categorized into a high RDW group. DM patients had poor survival rates when RAR or RDW values were greater than 3.22 or 13.3, respectively (**Figure 2B**).

## 4 Discussion

Here, we explored RAR in the context of DM epidemiology and long-term prognosis. Previous research reported that the combination of RDW and ALB serves as an indicator of RAR-related inflammation (19). Also a risk factor for DM, RAR is a novel inflammatory indicator. Importantly, DM is recognized to possess inflammatory pathologic characteristics. Pro-inflammatory cytokines (e.g. TNF-α) cause insufficient insulin secretion and resistance in DM patients (20). High blood sugar, in turn, affects hemodynamic parameters such as blood viscosity and promotes inflammation that damages red blood cells. As such, RAR reflects red blood cell status as well as systemic inflammation.

Inflammation and erythrocyte pathologies frequently interact. Inflammatory mediators promote damage to the endothelium as well as red blood cells and lead to atherosclerosis (21). Erythrocyte dysfunction also triggers inflammation (21, 22) and dysregulation in oxidation. Interestingly, RDW is known to be a biomarker for erythrocyte damage and assessing critical illness outcomes (23). Similarly, serum ALB is considered to be an inflammatory marker (24). A higher level of inflammation is suggested by a lower serum ALB level, especially in the setting of severe sepsis or septic shock (25). Because RAR is calculated from RDW and ALB, RAR can suggest the presence of erythrocyte dysfunction as well as inflammation.

For analysis of prognosis, RAR is considered in acute biliary pancreatitis (26), foot ulcers due to DM, and stroke. Importantly, RAR is determined using RDW and ALB values; RDW is utilized as a diagnostic indicator and prognosis biomarker for conditions such as atrial fibrillation (27), heart failure (28, 29) and viral infections (30), while RDW plays a significant role in cardiovascular and thrombotic pathology. Higher levels of RDW are associated with an increased risk of thrombotic disorders (31). The significant roles that RDW plays in cardiovascular and thrombotic pathologies highlight the importance of investigating RAR in detail. Meanwhile, higher levels of glycated albumin indicate either higher Glu levels (32) or lower serum ALB; serum ALB was previously reported to be a protective factor in DM prognosis (33). One study of a large Chinese cohort of 30,442 adults reported that the risk of type 2 DM significantly correlated with lower levels of ALB (34). In the context of prior literature having emphasized the crucial independent associations of RDW and ALB with DM, this study considered both to explore their combined use as a better predictive marker for DM.

In the adjusted Model 4, the RAR OR and RDW OR were 1.68 and 1.14, respectively, for DM prevalence. When adjusted for Q1, the Q4 RAR was greater than that of RDW for DM. As RAR levels increased, the prevalence of DM was also noted to rise. For the prognosis of all-cause mortality, RAR duplicated the result as the prevalence; values were significantly higher as compared to those of RDW. Furthermore, RCS was applied to explore the optimal threshold; the inverse L-curve for RAR revealed 3.22 to be the inflection point between the HR and DM (for RDW, it was 13.3). Importantly, DM patients with RAR values greater than 3.22 were likely to suffer a poor prognosis and shorter survival time as compared to those with RAR values ≤3.22. For RDW, DM patients with RDW values greater than 13.3 had a poorer prognosis as compared to those with RDW values ≤13.3. As such, an RAR of 3.22 was found to be optimal for determining whether the prognosis of DM patients was poor.

Further research is certainly warranted to further evaluate the association of RAR with DM. The association of RDW with short-term DM prognosis has been extensively studied, including outcomes at periods of 30 (35) and 90 (9–11) days. Here, the prognostic value of RAR was evaluated at over 84 months. The utilization of RAR in multiple clinical tests has demonstrated clear clinical value. As stated previously, RDW and ALB have already been validated as useful diagnostic indicators. Our findings warrant more detailed research concerning the clinical value of RAR. For instance, the prevalence of CKD, COPD, hypertension, ASCVD, and CHF were found to increase along with RAR (**Table 2**). As prevalence of the aforementioned conditions is rising, the diagnostic and prognostic values of RAR in respiratory, cardiovascular, metabolic, and urinary pathologies warrants further exploration. By considering RAR alongside other clinical factors, healthcare professionals will be better able to assess DM risk and prognosis, thereby leading to improved patient care and outcomes.

Here, we found significant disparities in sensitivity analysis for the risk of poor prognosis based on sex and age. The male DM patients had a higher risk of suffering poor prognosis as compared to female DM patients when RAR increased. Contrary to our conclusions, one study found that female patients with acute myocardial infarction were more sensitive to RAR (36). However, in a study of coronary artery disease (37) similar to ours result, the unadjusted RAR of male and <60 years coronary artery disease patients had a higher risk of carotid plaque than females. This sex difference may be triggered by the severity of the disease, females’ estrogen level, and menstruation. Acute myocardial infarction (38) is more urgent and fatal than DM which may trigger the sex difference of RAR. Due to menstruation and estrogen, females are more prone to anemia (39) and increased RDW which results in the RAR level being higher than males. Since male RAR is more stable, males are more sensitive to slight fluctuations in RAR.

The advantages of this study included a combination of cross-sectional and prospective analyses, a large sample size and a long follow-up duration, as well as numerous covariates applied in model calibration. First, a cross-sectional approach was employed to for prevalence analysis, while prospective evaluation was performed in the context of a survival analysis. Second, this study analyzed data from over 40,000 individuals compiled over the course of over two decades (i.e. 1999–2020). Finally, more than 30 covariates were adjusted for to ensure result validity. This study ultimately aimed to shed light on the association between RAR and DM in terms of incidence and prognosis.

This study was not without limitations. First, our results should be treated cautiously, as definite causality cannot be established. Further in-depth research to validate the clinical relevance of RAR to DM is certainly required. Although many covariates were modulated, potential confounders likely affected our findings. Second, data misclassification may have led to underestimates of RAR values (40), thereby affecting finding accuracy. Nevertheless, our research highlighted the valuable association between RAR and DM.

In conclusion, future research to investigate the relationship between inflammation and RAR is warranted. The significant role of RDW in cardiovascular and thrombotic disorders as well as the association of RDW with such pathologies underscores the potential clinical importance of RAR.

## 5 Conclusion

Here, we found that DM prevalence is significantly associated with greater RAR values. Furthermore, greater RAR values were found to associate with worse prognosis in DM patients. Importantly, the risk of poor prognosis was found to be highest among female DM patients ≤60 years of age. As such, further research concerning RAR as an independent risk factor predictive for DM prevalence and poor prognosis is warranted.

## Data availability statement

The original contributions presented in the study are included in the article/**Supplementary Material**. Further inquiries can be directed to the corresponding author.

## Ethics statement

The studies involving humans were approved by The National Center for Health Statistics Ethics Review Committee. The studies were conducted in accordance with the local legislation and institutional requirements. The participants provided their written informed consent to participate in this study. Written informed consent was obtained from the individual(s) for the publication of any potentially identifiable images or data included in this article.

## Author contributions

JL: Writing – original draft. XW: Writing – review & editing. TG: Writing – review & editing, Conceptualization. QZ: Writing – review & editing, Methodology, Data curation. SZ: Writing – review & editing, Formal analysis, Conceptualization. YX: Writing – review & editing, Investigation. WY: Writing – review & editing, Investigation, Data curation, Formal analysis. ZY: Writing – review & editing, Methodology, Formal analysis. HY: Writing – review & editing, Supervision, Software.

## References

[1] Unnikrishnan R Anjana RM Mohan V. Diabetes mellitus and its complications in India. Nat Rev Endocrinol. (2016) 12:357–70. doi: 10.1038/nrendo.2016.53 27080137

[2] American Diabetes Association. Diagnosis and classification of diabetes mellitus. Diabetes Care. (2011) 34 Suppl 1:S62–69. doi: 10.2337/dc11-S062 PMC3006051 21193628

[3] Knychala MA Garrote-Filho MDS Batista da Silva B Neves de Oliveira S Yasminy Luz S Marques Rodrigues MO. Red cell distribution width and erythrocyte osmotic stability in type 2 diabetes mellitus. J Cell Mol Med. (2021) 25:2505–16. doi: 10.1111/jcmm.16184 PMC7933938 33591627

[4] Gajecki D Gawryś J Wiśniewski J Fortuna P Szahidewicz-Krupska E Doroszko A. A cross-talk between the erythrocyte L-arginine/ADMA/nitric oxide metabolic pathway and the endothelial function in subjects with type 2 diabetes mellitus. Nutrients. (2021) 13:2306. doi: 10.3390/nu13072306 34371816 PMC8308357

[5] Zhou Z Collado A Sun C Tratsiakovich Y Mahdi A Winter H. Downregulation of erythrocyte miR-210 induces endothelial dysfunction in type 2 diabetes. Diabetes. (2022) 71:285–97. doi: 10.2337/db21-0093 34753800

[6] Williams A Bissinger R Shamaa H Patel S Bourne L Artunc F. Pathophysiology of red blood cell dysfunction in diabetes and its complications. Pathophysiology. (2023) 30:327–45. doi: 10.3390/pathophysiology30030026 PMC10443300 37606388

[7] Wang J Zhang Y Wan Y Fan Z Xu R. The relationship between red blood cell distribution width and incident diabetes in Chinese adults: A cohort study. J Diabetes Res. (2020) 2020:1623247. doi: 10.1155/2020/1623247 32185232 PMC7063217

[8] Jie Chee Y Seneviratna A Joo Lim C Chiong CX Peh DS Hawkins R. Red cell distribution width is associated with mortality and cardiovascular complications in diabetes mellitus in Singapore. Eur J Prev Cardiol. (2020) 27:216–9. doi: 10.1177/2047487319836854 31130002

[9] Ni Q Wang X Wang J Chen P. The red blood cell distribution width-albumin ratio: A promising predictor of mortality in heart failure patients - A cohort study. Clin Chim Acta. (2022) 527:38–46. doi: 10.1016/j.cca.2021.12.027 34979101

[10] Xu W Huo J Chen G Yang K Huang Z Peng L. Association between red blood cell distribution width to albumin ratio and prognosis of patients with sepsis: A retrospective cohort study. Front Nutr. (2022) 9:1019502. doi: 10.3389/fnut.2022.1019502 36211519 PMC9539557

[11] Seo YJ Yu J Park JY Lee N Lee J Park JH. Red cell distribution width/albumin ratio and 90-day mortality after burn surgery. Burns Trauma. (2022) 10:tkab050. doi: 10.1093/burnst/tkab050 35097135 PMC8793164

[12] Zhao F Liu M Kong L. Association between red blood cell distribution width-to-albumin ratio and diabetic retinopathy. J Clin Lab Anal. (2022) 36:e24351. doi: 10.1002/jcla.24351 35285094 PMC8993659

[13] Hong J Hu X Liu W Qian X Jiang F Xu Z. Impact of red cell distribution width and red cell distribution width/albumin ratio on all-cause mortality in patients with type 2 diabetes and foot ulcers: a retrospective cohort study. Cardiovasc Diabetol. (2022) 21:91. doi: 10.1186/s12933-022-01534-4 35658957 PMC9166463

[14] Palmer MK Toth PP. Trends in lipids, obesity, metabolic syndrome, and diabetes mellitus in the United States: an NHANES analysis, (2003-2004 to 2013-2014). Obes (Silver Spring). (2019) 27:309–14. doi: 10.1002/oby.22370 30677260

[15] Feng M McSparron JI Kien DT Stone DJ Roberts DH Schwartzstein RM. Transthoracic echocardiography and mortality in sepsis: analysis of the MIMIC-III database. Intensive Care Med. (2018) 44:884–92. doi: 10.1007/s00134-018-5208-7 29806057

[16] Zhang Q Xiao S Jiao X Shen Y. The triglyceride-glucose index is a predictor for cardiovascular and all-cause mortality in CVD patients with diabetes or pre-diabetes: evidence from NHANES 2001-2018. Cardiovasc Diabetol. (2023) 22:279. doi: 10.1186/s12933-023-02030-z 37848879 PMC10583314

[17] Whelton PK Carey RM Aronow WS Casey DE Jr. Collins KJ Dennison Himmelfarb C. 2017 ACC/AHA/AAPA/ABC/ACPM/AGS/APhA/ASH/ASPC/NMA/PCNA guideline for the prevention, detection, evaluation, and management of high blood pressure in adults: A report of the American college of cardiology/American heart association task force on clinical practice guidelines. Hypertension. (2018) 71:e13–e115. doi: 10.1161/HYP.0000000000000066 29133356

[18] Liu X Zhang D Liu Y Sun X Han C Wang B. Dose-response association between physical activity and incident hypertension: A systematic review and meta-analysis of cohort studies. Hypertension. (2017) 69:813–20. doi: 10.1161/HYPERTENSIONAHA.116.08994 28348016

[19] Zhou D Wang J Li X. The red blood cell distribution width-albumin ratio was a potential prognostic biomarker for diabetic ketoacidosis. Int J Gen Med. (2021) 14:5375–80. doi: 10.2147/IJGM.S327733 PMC8434876 34522133

[20] Bendek MJ Canedo-Marroquín G Realini O Retamal IN Hernández M Hoare A. Periodontitis and gestational diabetes mellitus: A potential inflammatory vicious cycle. Int J Mol Sci. (2021) 22:11831. doi: 10.3390/ijms222111831 34769262 PMC8584134

[21] Robbie L Libby P. Inflammation and atherothrombosis. Ann N Y Acad Sci. (2001) 947:167–179;discussion 179-180. doi: 10.1111/j.1749-6632.2001.tb03939.x 11795264

[22] Toledo SLO Guedes JVM Alpoim PN Rios DRA Pinheiro MB. Sickle cell disease: Hemostatic and inflammatory changes, and their interrelation. Clin Chim Acta. (2019) 493:129–37. doi: 10.1016/j.cca.2019.02.026 30825426

[23] Said AS Spinella PC Hartman ME Steffen KM Jackups R Holubkov R. RBC distribution width: biomarker for red cell dysfunction and critical illness outcome? Pediatr Crit Care Med. (2017) 18:134–42. doi: 10.1097/PCC.0000000000001017 PMC5291765 27832023

[24] Eckart A Struja T Kutz A Baumgartner A Baumgartner T Zurfluh S. Relationship of nutritional status, inflammation, and serum albumin levels during acute illness: A prospective study. Am J Med. (2020) 133:713–722.e717. doi: 10.1016/j.amjmed.2019.10.031 31751531

[25] Caironi P Tognoni G Masson S Fumagalli R Pesenti A Romero M. Albumin replacement in patients with severe sepsis or septic shock. N Engl J Med. (2014) 370:1412–21. doi: 10.1056/NEJMoa1305727 24635772

[26] Donmez M Ayata O. Prognostic significance of the red cell distribution width/albumin ratio in the prediction of the severity of acute biliary pancreatitis: A preliminary report. Cureus. (2022) 14:e30183. doi: 10.7759/cureus.30183 36238420 PMC9552954

[27] Wang Z Korantzopoulos P Roever L Liu T. Red blood cell distribution width and atrial fibrillation. biomark Med. (2020) 14:1289–98. doi: 10.2217/bmm-2020-0041 33021384

[28] Xanthopoulos A Giamouzis G Dimos A Skoularigki E Starling RC Skoularigis J. Red blood cell distribution width in heart failure: pathophysiology, prognostic role, controversies and dilemmas. J Clin Med. (2022) 11:1951. doi: 10.3390/jcm11071951 35407558 PMC8999162

[29] Zhang L Lin Y Wang K Han L Zhang X Gao X. Multiple-model machine learning identifies potential functional genes in dilated cardiomyopathy. Front Cardiovasc Med. (2022) 9:1044443. doi: 10.3389/fcvm.2022.1044443 36712235 PMC9874116

[30] Owoicho O Tapela K Olwal CO Djomkam Zune AL Nganyewo NN Quaye O. Red blood cell distribution width as a prognostic biomarker for viral infections: prospects and challenges. biomark Med. (2022) 16:41–50. doi: 10.2217/bmm-2021-0364 34784758 PMC8597662

[31] Montagnana M Cervellin G Meschi T Lippi G. The role of red blood cell distribution width in cardiovascular and thrombotic disorders. Clin Chem Lab Med. (2011) 50:635–41. doi: 10.1515/cclm.2011.831 22505527

[32] Park S Lee W Chung HS Hong KS. Diagnostic utility of serum glycated albumin for diabetes mellitus and its correlation with hyperlipidemia. Ann Lab Med. (2016) 36:306–12. doi: 10.3343/alm.2016.36.4.306 PMC4855049 27139602

[33] Wang N Xu Z Han P Li T. Glycated albumin and ratio of glycated albumin to glycated hemoglobin are good indicators of diabetic nephropathy in type 2 diabetes mellitus. Diabetes Metab Res Rev. (2017) 33:2843. doi: 10.1002/dmrr.2843 27537245

[34] Hu F Lou Y Shi J Cao L Wang C Ma J. Baseline serum albumin and its dynamic change is associated with type 2 diabetes risk: A large cohort study in China. Diabetes Metab Res Rev. (2020) 36:e3296. doi: 10.1002/dmrr.3296 32017334

[35] Lu C Long J Liu H Xie X Xu D Fang X. Red blood cell distribution width-to-albumin ratio is associated with all-cause mortality in cancer patients. J Clin Lab Anal. (2022) 36:e24423. doi: 10.1002/jcla.24423 35396747 PMC9102686

[36] Li D Ruan Z Wu B. Association of red blood cell distribution width-albumin ratio for acute myocardial infarction patients with mortality: A retrospective cohort study. Clin Appl Thromb Hemost. (2022) 28:10760296221121286. doi: 10.1177/10760296221121286 36045634 PMC9445528

[37] Huang M Liu F Li Z Liu Y Su J Ma M. Relationship between red cell distribution width/albumin ratio and carotid plaque in different glucose metabolic states in patients with coronary heart disease: a RCSCD-TCM study in China. Cardiovasc Diabetol. (2023) 22:39. doi: 10.1186/s12933-023-01768-w 36814226 PMC9948352

[38] Zhang L Liu Y Wang K Ou X Zhou J Zhang H. Integration of machine learning to identify diagnostic genes in leukocytes for acute myocardial infarction patients. J Transl Med. (2023) 21:761. doi: 10.1186/s12967-023-04573-x 37891664 PMC10612217

[39] Abdelrahman EG Gasim GI Musa IR Elbashir LM Adam I. Red blood cell distribution width and iron deficiency anemia among pregnant Sudanese women. Diagn Pathol. (2012) 7:168. doi: 10.1186/1746-1596-7-168 23206545 PMC3538607

[40] Wang Y Fang Y Magliano DJ Charchar FJ Sobey CG Drummond GR. Fasting triglycerides are positively associated with cardiovascular mortality risk in people with diabetes. Cardiovasc Res. (2023) 119:826–34. doi: 10.1093/cvr/cvac124 PMC10153411 35905014

