# Supplementary material for: Red blood cell distribution width to albumin ratio associates with prevalence and long-term diabetes mellitus prognosis: an overview of NHANES 1999–2020 data
Source: Front Endocrinol (Lausanne). 2024 Jul 24;15:1362077. doi: 10.3389/fendo.2024.1362077 (PMC11303207; doi:10.3389/fendo.2024.1362077)
Supplement: Supplementary file 1 [file DataSheet_1.docx]

**Table S1.** The General characteristics for female and male in NHANES.

| **Variable** | **Female (*n*=21,630)** | **Male (*n*=18,928)** | **P_value** |
| --- | --- | --- | --- |
| DM, % | 4882(16.65) | 5272(20.20) | < 0.0001 |
| RAR, mL/g | 3.17±0.01 | 2.96±0.01 | < 0.0001 |
| CKD, % | 3873(14.99) | 3497(12.98) | < 0.0001 |
| COPD, % | 549(3.10) | 686(3.62) | 0.04 |
| Hypertension, % | 7676(31.28) | 7421(34.02) | < 0.0001 |
| ASCVD, % | 1675(6.55) | 2250(8.77) | < 0.0001 |
| Anemia, % |  |  | < 0.0001 |
| Mild | 1679(5.66) | 1167(3.51) |  |
| Moderate | 802(2.61) | 188(0.56) |  |
| Non-Anaemia | 19104(91.58) | 17558(95.91) |  |
| Severe | 45(0.15) | 15(0.02) |  |
| CHF, % | 569(2.00) | 755(2.70) | < 0.0001 |
| Ethnicity, % |  |  | < 0.0001 |
| Mexican American | 3917(7.88) | 3313(8.69) |  |
| Non-Hispanic Black | 4412(10.99) | 3815( 9.48) |  |
| Non-Hispanic White | 9327(68.36) | 8464(69.27) |  |
| Other Hispanic | 1904(6.00) | 1479(5.76) |  |
| Other Race | 2070(6.78) | 1857(6.80) |  |
| Age, year | 44.95±0.20 | 44.19±0.21 | < 0.0001 |
| LymP, % | 30.23±0.09 | 29.62±0.09 | < 0.0001 |
| MonP, % | 7.52±0.02 | 8.41±0.03 | < 0.0001 |
| SegneP, % | 59.04±0.10 | 58.38±0.09 | < 0.0001 |
| EoP, % | 2.56±0.02 | 2.95±0.02 | < 0.0001 |
| BaP, % | 0.72±0.01 | 0.70±0.01 | 0.02 |
| Lym, 1000 cells/μL | 2.17±0.01 | 2.09±0.01 | < 0.0001 |
| Mon, 1000 cells/μL | 0.54±0.00 | 0.59±0.00 | < 0.0001 |
| Eo, 1000 cells/μL | 0.19±0.00 | 0.21±0.00 | < 0.0001 |
| Ba, 1000 cells/μL | 0.05±0.00 | 0.04±0.00 | < 0.001 |
| RBC, million cells/μL | 4.44±0.01 | 4.96±0.01 | < 0.0001 |
| Hg, g/dl | 13.43±0.02 | 15.19±0.02 | < 0.0001 |
| Hem, % | 39.64±0.06 | 44.58±0.06 | < 0.0001 |
| MCV, fL | 89.45±0.07 | 90.11±0.07 | < 0.0001 |
| MCH, pg | 30.31±0.03 | 30.71±0.03 | < 0.0001 |
| MCHC, g/cL | 33.87±0.03 | 34.08±0.03 | < 0.0001 |
| RDW, % | 13.11±0.02 | 12.89±0.01 | < 0.0001 |
| Plt, 1000 cells/μL | 266.22±0.81 | 238.40±0.71 | < 0.0001 |
| MPV, fL | 8.22±0.01 | 8.16±0.01 | < 0.0001 |
| ALB, g/dL | 4.17±0.00 | 4.40±0.01 | < 0.0001 |
| ALT, U/L | 20.32±0.10 | 29.37±0.23 | < 0.0001 |
| AST, U/L | 22.55±0.11 | 26.91±0.17 | < 0.0001 |
| Ca, mg/dL | 9.38±0.01 | 9.47±0.01 | < 0.0001 |
| HCO3, mmol/L | 24.43±0.05 | 25.17±0.05 | < 0.0001 |
| GGT, U/L | 22.77±0.31 | 33.52±0.43 | < 0.0001 |
| Glu, mg/dL | 96.04±0.30 | 101.28±0.38 | < 0.0001 |
| TP, g/dL | 7.11±0.01 | 7.22±0.01 | < 0.0001 |
| TG, mg/dL | 128.24±1.01 | 165.87±1.76 | < 0.0001 |
| UA, mg/dL | 4.64±0.01 | 6.00±0.01 | < 0.0001 |
| Na, mmol/L | 139.10±0.06 | 139.50±0.06 | < 0.0001 |
| Cl, mmol/L | 103.32±0.06 | 102.73±0.06 | < 0.0001 |

**Table S2.** The General characteristics for individuals with age <= 60 or >60 years in NHANES.

| **Variable** | **Age <=60 (*n*=29,303)** | **Age >60 (*n*=11,253)** | **P_value** |
| --- | --- | --- | --- |
| DM, % | 4242(11.66) | 5912(44.91) | < 0.0001 |
| RAR, mL/g | 3.03±0.00 | 3.26±0.01 | < 0.0001 |
| CKD, % | 2740( 8.11) | 4630(37.99) | < 0.0001 |
| COPD, % | 470(2.01) | 765(8.67) | < 0.0001 |
| Hypertension, % | 7069(23.56) | 8028(68.56) | < 0.0001 |
| ASCVD, % | 1094( 3.33) | 2831(24.19) | < 0.0001 |
| Anemia, % |  |  |  |
| Mild | 1454(3.57) | 1392(8.99) |  |
| Moderate | 616(1.45) | 374(2.45) |  |
| Non-Anaemia | 27187(94.88) | 9475(88.51) |  |
| Severe | 48(0.10) | 12(0.05) | < 0.0001 |
| CHF, % | 338(0.88) | 986(7.99) | < 0.0001 |
| Ethnicity, % |  |  |  |
| Mexican American | 5511(9.24) | 1719(4.32) |  |
| Non-Hispanic Black | 6083(10.81) | 2144( 8.18) |  |
| Non-Hispanic White | 12026(66.32) | 5765(78.65) |  |
| Other Hispanic | 2547(6.47) | 836(3.53) |  |
| Other Race | 3138(7.16) | 789(5.31) | < 0.0001 |
| Sex, (Male), % |  |  | < 0.0001 |
| Age, year | 38.06±0.14 | 70.72±0.09 | < 0.0001 |
| LymP, % | 30.46±0.08 | 27.88±0.12 | < 0.0001 |
| MonP, % | 7.80±0.02 | 8.47±0.03 | < 0.0001 |
| SegneP, % | 58.42±0.09 | 59.95±0.13 | < 0.0001 |
| EoP, % | 2.68±0.01 | 3.01±0.02 | < 0.0001 |
| BaP, % | 0.70±0.00 | 0.75±0.01 | < 0.0001 |
| Lym, 1000 cells/μL | 2.17±0.01 | 1.98±0.02 | < 0.0001 |
| Mon, 1000 cells/μL | 0.56±0.00 | 0.59±0.00 | < 0.0001 |
| Eo, 1000 cells/μL | 0.19±0.00 | 0.21±0.00 | < 0.0001 |
| Ba, 1000 cells/μL | 0.04±0.00 | 0.05±0.00 | < 0.0001 |
| RBC, million cells/μL | 4.72±0.01 | 4.53±0.01 | < 0.0001 |
| Hg, g/dl | 14.33±0.02 | 13.95±0.03 | < 0.0001 |
| Hem, % | 42.13±0.06 | 41.22±0.07 | < 0.0001 |
| MCV, fL | 89.39±0.07 | 91.23±0.09 | < 0.0001 |
| MCH, pg | 30.41±0.03 | 30.87±0.04 | < 0.0001 |
| MCHC, g/cL | 34.01±0.02 | 33.82±0.03 | < 0.0001 |
| RDW, % | 12.90±0.01 | 13.43±0.02 | < 0.0001 |
| Plt, 1000 cells/μL | 257.37±0.71 | 236.60±1.06 | 0.24 |
| MPV, fL | 8.19±0.01 | 8.21±0.02 | < 0.0001 |
| ALB, g/dL | 4.31±0.00 | 4.16±0.01 | < 0.0001 |
| ALT, U/L | 25.24±0.16 | 21.80±0.16 | 0.35 |
| AST, U/L | 24.63±0.12 | 24.46±0.14 | 0.001 |
| Ca, mg/dL | 9.42±0.01 | 9.44±0.01 | < 0.0001 |
| HCO3, mmol/L | 24.64±0.05 | 25.33±0.05 | 0.02 |
| GGT, U/L | 27.51±0.31 | 28.96±0.51 | < 0.0001 |
| Glu, mg/dL | 94.94±0.26 | 112.69±0.56 | < 0.0001 |
| TP, g/dL | 7.19±0.01 | 7.04±0.01 | < 0.0001 |
| TG, mg/dL | 142.97±1.24 | 157.28±1.44 | < 0.0001 |
| UA, mg/dL | 5.19±0.01 | 5.61±0.02 | < 0.0001 |
| Na, mmol/L | 139.21±0.06 | 139.56±0.08 | < 0.0001 |
| Cl, mmol/L | 103.17±0.06 | 102.53±0.07 |  |

**Table S3.** The univariate Logistic result

| **Variables** | **Univariate Logistic regression** | |
| --- | --- | --- |
|  | **OR (95% CI)** | **P_value** |
| Age | 1.07(1.07,1.07) | <0.0001 |
| LymP | 0.98(0.97,0.98) | <0.0001 |
| SegneP | 1.02(1.01,1.02) | <0.0001 |
| EoP | 1.05(1.04,1.07) | <0.0001 |
| BaP | 1.13(1.06,1.21) | <0.001 |
| Lym | 1.07(1.02,1.12) | 0.004 |
| Mon | 2.83(2.45,3.28) | <0.0001 |
| Eo | 3.11(2.59,3.74) | <0.0001 |
| Ba | 24.47(13.75,43.52) | <0.0001 |
| Hg | 0.91(0.90,0.93) | <0.0001 |
| Hem | 0.98(0.98,0.99) | <0.0001 |
| MCV | 0.97(0.97,0.98) | <0.0001 |
| MCH | 0.91(0.90,0.92) | <0.0001 |
| MCHC | 0.77(0.74,0.81) | <0.0001 |
| RDW | 1.42(1.38,1.46) | <0.0001 |
| Plt | 1.00(1.00,1.00) | <0.0001 |
| MPV | 1.16(1.12,1.20) | <0.0001 |
| ALB | 0.25(0.23,0.28) | <0.0001 |
| ALT | 1.01(1.01,1.01) | <0.0001 |
| AST | 1.01(1.00,1.01) | <0.0001 |
| HCO3 | 1.04(1.03,1.06) | <0.0001 |
| GGT | 1.01(1.00,1.01) | <0.0001 |
| Glu | 1.10(1.09,1.10) | <0.0001 |
| TP | 0.85(0.80,0.92) | <0.0001 |
| TG | 1.00(1.00,1.00) | <0.0001 |
| UA | 1.30(1.27,1.33) | <0.0001 |
| Na | 0.95(0.94,0.97) | <0.0001 |
| Cl | 0.87(0.86,0.88) | <0.0001 |
| RAR | 3.05(2.85,3.27) | <0.0001 |
| CKD |  |  |
| no | ref | ref |
| yes | 6.12(5.68,6.60) | <0.0001 |
| COPD |  |  |
| no | ref | ref |
| yes | 3.06(2.66,3.52) | <0.0001 |
| Hypertension |  |  |
| no | ref | ref |
| yes | 6.85(6.35,7.38) | <0.0001 |
| ASCVD |  |  |
| no | ref | ref |
| yes | 6.48(5.89,7.13) | <0.0001 |
| anemia |  |  |
| Mild | ref | ref |
| Moderate | 0.89(0.71,1.11) | 0.30 |
| Non-Anaemia | 0.37(0.33,0.42) | <0.0001 |
| Severe | 0.16(0.08,0.34) | <0.0001 |
| CHF |  |  |
| no | ref | ref |
| yes | 8.80(7.51,10.31) | <0.0001 |
| Sex |  |  |
| Female | ref | ref |
| Male | 1.27(1.19,1.35) | <0.0001 |
| Ethnicity |  |  |
| Mexican American | ref | ref |
| Non-Hispanic Black | 1.26(1.11,1.43) | <0.001 |
| Non-Hispanic White | 0.76(0.68,0.85) | <0.0001 |
| Other Hispanic | 0.91(0.79,1.05) | 0.19 |
| Other Race | 1.18(1.03,1.37) | 0.02 |

**Table S4.** The association of DM in RAR with stratification of sex.

| **Sex** | **Variables** | **DM** | | | | | | | |
| --- | --- | --- | --- | --- | --- | --- | --- | --- | --- |
|  |  | **OR (95% CI)^a^** | **P-value** | **OR (95% CI)^b^** | **P-value** | **OR (95% CI)^c^** | **P-value** | **OR (95% CI)^d^** | **P-value** |
| Male | RAR | 6.92(5.99,8.00) | <0.0001 | 2.88(2.49,3.34) | <0.0001 | 2.23(1.85,2.68) | <0.0001 | 1.26(0.79, 2.00) | 0.33 |
|  | Q1 | ref | ref | ref | ref | ref | ref | ref | ref |
|  | Q2 | 2.33(2.01, 2.71) | 0.001 | 1.55(1.32,1.82) | <0.0001 | 1.48(1.25,1.77) | <0.0001 | 1.32(1.03, 1.70) | 0.03 |
|  | Q3 | 4.40(3.83, 5.04) | <0.0001 | 2.25(1.91,2.64) | <0.0001 | 1.96(1.65,2.33) | <0.0001 | 1.44(1.03, 2.00) | 0.03 |
|  | Q4 | 10.23(8.79,11.91) | <0.0001 | 3.81(3.21,4.52) | <0.0001 | 2.65(2.16,3.25) | <0.0001 | 1.74(1.08, 2.81) | 0.02 |
| Female | RAR | 2.50(2.30,2.71) | <0.0001 | 2.48(2.25,2.72) | <0.0001 | 2.13(1.85,2.46) | <0.0001 | 0.94(0.75, 1.18) | 0.59 |
|  | Q1 | ref | ref | ref | ref | ref | ref | ref | ref |
|  | Q2 | 1.85(1.59,2.16) | <0.0001 | 1.46(1.24,1.73) | <0.0001 | 1.33(1.11,1.60) | 0.002 | 1.18(0.92, 1.51) | 0.18 |
|  | Q3 | 3.44(3.00,3.94) | <0.0001 | 2.56(2.20,2.98) | <0.0001 | 2.17(1.84,2.57) | <0.0001 | 1.78(1.35, 2.35) | <0.0001 |
|  | Q4 | 6.24(5.39,7.24) | <0.0001 | 4.99(4.26,5.84) | <0.0001 | 3.65(3.03,4.41) | <0.0001 | 2.72(1.88, 3.93) | <0.0001 |

a Model 1: unadjusted; b Model 2: adjusted with Age; c Model 3: adjusted Age, CKD, COPD, Hypertension, ASCVD, Anemia, CHF, Ethnicity; d Model 4: adjusted with Age, CKD, COPD, Hypertension, ASCVD, Anemia, CHF, Ethnicity, LymP, SegneP, EoP, BaP, Lym, Mon, Eo, Ba, MCV, MCH, MCHC, Plt, MPV, ALT, AST, HCO3, GGT, Glu, TP, TG, UA, Na, and Cl.

**Table S5.** The association of DM in RAR with stratification of age.

| **Age** | **Variables** | **DM** | | | | | | | |
| --- | --- | --- | --- | --- | --- | --- | --- | --- | --- |
|  |  | **OR (95% CI)^a^** | **P-value** | **OR (95% CI)^b^** | **P-value** | **OR (95% CI)^c^** | **P-value** | **OR (95% CI)^d^** | **P-value** |
| >60 | RAR | 2.34(2.07,2.65) | <0.0001 | 2.43(2.13,2.76) | <0.0001 | 1.76(1.51,2.05) | <0.0001 | 1.10(0.78, 1.54) | 0.58 |
|  | Q1 | ref | ref | ref | ref | ref | ref | ref | ref |
|  | Q2 | 1.24(1.05,1.47) | 0.29 | 1.27(1.07,1.50) | 0.01 | 1.15(0.96,1.38) | 0.14 | 1.12(0.87, 1.43) | 0.37 |
|  | Q3 | 1.66(1.40,1.97) | <0.001 | 1.72(1.45,2.05) | <0.0001 | 1.52(1.25,1.85) | <0.0001 | 1.33(0.99, 1.79) | 0.06 |
|  | Q4 | 2.81(2.41,3.27) | <0.0001 | 2.96(2.53,3.47) | <0.0001 | 2.02(1.68,2.44) | <0.0001 | 1.49(1.00, 2.20) | 0.05 |
| <=60 | RAR | 2.60(2.39,2.83) | <0.0001 | 3.11(2.84,3.41) | <0.0001 | 2.65(2.28,3.07) | <0.0001 | 0.92(0.72, 1.17) | 0.48 |
|  | Q1 | ref | ref | ref | ref | ref | ref | ref | ref |
|  | Q2 | 1.81(1.58,2.07) | 0.002 | 2.01(1.75,2.30) | <0.0001 | 1.73(1.49,2.00) | <0.0001 | 1.41(1.12, 1.77) | 0.004 |
|  | Q3 | 3.15(2.77,3.58) | <0.0001 | 3.83(3.37,4.35) | <0.0001 | 2.89(2.52,3.32) | <0.0001 | 1.91(1.48, 2.48) | <0.0001 |
|  | Q4 | 5.62(4.89,6.46) | <0.0001 | 7.78(6.77,8.96) | <0.0001 | 4.99(4.21,5.90) | <0.0001 | 3.64(2.57, 5.14) | <0.0001 |

a Model 1: unadjusted; b Model 2: adjusted with Sex; c Model 3: adjusted with Sex, CKD, COPD, Hypertension, ASCVD, Anemia, CHF, Ethnicity; d Model 4: adjusted with Sex, CKD, COPD, Hypertension, ASCVD, Anemia, CHF, Ethnicity, LymP, SegneP, EoP, BaP, Lym, Mon, Eo, Ba, MCV, MCH, MCHC, Plt, MPV, ALT, AST, HCO3, GGT, Glu, TP, TG, UA, Na, and Cl.
